# Metallization on Sapphire and Low-Temperature Joining with Metal Substrates

**DOI:** 10.3390/ma15051783

**Published:** 2022-02-26

**Authors:** Jiajun Fang, Qiaoxin Zhang, Zhou Luo, Wei Huang, Zhenyu Liu, Zhiwen Chen, Xueqiang Cao, Li Liu

**Affiliations:** 1 School of Materials Science and Engineering, Wuhan University of Technology, Wuhan 430070, China; jjf@whut.edu.cn (J.F.); zhangqx@whut.edu.cn (Q.Z.); lz15571339343@whut.edu.cn (Z.L.); whuthuangwei@whut.edu.cn (W.H.); liu.zy@whut.edu.cn (Z.L.); xcao@whut.edu.cn (X.C.); 2 The Institute of Technological Science, Wuhan University, Wuhan 430072, China; zwchen_lu@163.com

**Keywords:** sapphire, surface metallization, deposition mechanism, wettability, low-temperature joining

## Abstract

To meet the packaging requirements of sapphire in special electronic components, there is an urgent need for a joining process that can realize a good connection between sapphire and dissimilar metals at a low temperature. In this work, the surface of a sapphire substrate was successfully catalytically activated and metallized by an electroless nickel plating process. Moreover, the solderability and interconnection of metallized sapphire with Sn-based solders were evaluated and investigated at 250 °C, and the wetting angle of the Sn-based solders on sapphire on sapphire without and with metallization was 125° and 51°, respectively. The interfacial microscopic morphology and element distribution in the Cu/Sn-Ag solder/sapphire solder joints were analyzed. It was found that the middle solder layer has diffused during the reflow process, inferring good adhesion between sapphire and Cu substrate with the aid of the Ni-P deposition. Thus, a sapphire welding method with a simple process suitable for practical applications is demonstrated.

## 1. Introduction

Sapphire has superior optical and mechanical properties, including high melting point, high hardness, strong corrosion resistance and good thermal conductivity, which makes sapphire highly demanded in electronics and scientific instruments [1,2]. For electronics, sapphire is required to achieve joining with common metals by a simple reflow process. Unfortunately, sapphire is hard to achieve reliable solder joints during the reflow process as it is an Al_2_O_3_ single crystal. Currently, enormous works have been conducted on sapphire welding by various processes. Metallization on the sapphire surface is an important method. Related research on metallization methods of sapphire is listed in Table 1. Another method is using solders with active additions of Ti, Al and Mg. For instance, Ti addition in solders can significantly promote the solder wettability on sapphire. Mu et al. systematically studied the wetting behaviors of a Sn-Ti alloy on sapphire by the solid drop method [3]. However, Ti-based solders were required to be brazed in a high-temperature vacuum environment, which has been reported by Ning et al. [4]. With the assistance of ultrasonic, the brazing temperature of sapphire solder joints can be greatly reduced. Cui et al. used an Al-4.5Cu-1.5Mg alloy as a filling metal to connect sapphire through ultrasonic-assisted hot dipping [5]. A dense transient layer formed at the metal–sapphire interface to achieve solid connections between sapphire and the aluminum alloy. At present, various related researches on sapphire welding have been conducted in the above aspects. Unfortunately, they all have shortcomings for electronics applications, including high joining temperature, specific active solder, auxiliary ultrasonic brazing and so on. 

Electroless nickel plating is a conventional surface treatment technology for wide applications prospects [10,11,12,13,14], which has the advantages of uniform deposition, dense plating, good conductivity and good solderability [15,16]. Electroless nickel plating on porous alumina ceramics was achieved by Zhang et al. [17]. Dehchar et al. successfully deposited copper films on non-conductive epoxy glass substrates by electroless plating as well [18]. Currently, many works related to electroless nickel plating have been carried out on different ceramic substrates. However, the related research on electroless nickel plating on sapphire substrates has not been reported yet.

In this work, we realized the metallization on sapphire by electroless nickel plating and elaborated its deposition mechanism. Therefore, the Cu/solder/sapphire sandwich solder joints can be fabricated, and then the interfacial morphology of these solder joints can be observed. Moreover, the fracture mechanism of solder joints was also studied. Finally, sapphire substrates can be joined by a simple reflow process at a low temperature and low pressure.

## 2. Experimental Works

In this work, sapphire substrates (10 mm × 10 mm × 1 mm, α-Al_2_O_3_ single crystal, purity >99.9%) were used for electroless plating. To achieve electroless nickel plating on the sapphire surface, the influence of substrate pretreatment on sapphire was studied to successfully deposit electroless nickel plating. The surface roughness of the sapphire substrates was measured by an atomic force microscope (AFM, Nanoscope IV, Veeco, Suzhou, China). Different types of chemical etching solutions were chosen to prepare the smooth deposition of the coatings. After grinding, etching, sensitizing, activating and initial plating (25 s) of the sapphire substrates, the surface morphology of sapphire after each process was observed through a scanning electron microscope (SEM, MIRA 3, TESCAN, Shanghai, China). Moreover, the changes in the surface morphology of the substrates were compared and analyzed.

The electroless plating solution is an acidic solution composed of deionized water, nickel sulfate, sodium hypophosphite, sodium citrate and ammonium sulfate. Table 2 lists the chemical reagents and the corresponding electroless plating conditions. The crystal structure of Ni-P coating was also analyzed by X-ray diffraction (XRD, Smart SE, Rigaku, Tokyo, Japan). Afterwards, the surface morphology and chemical composition of the electroless deposition by SEM and EDS (MIRA 3, TESCAN, Shanghai, China), respectively.

**Figure 1:**
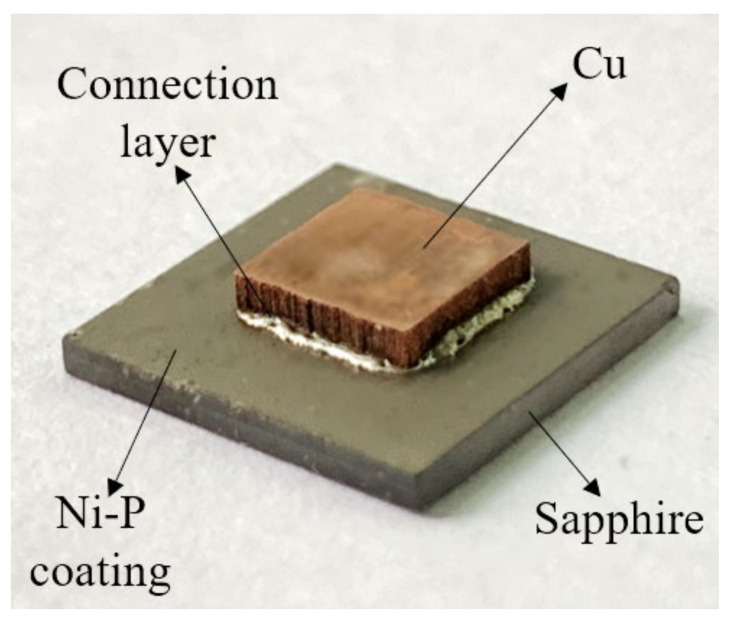
Macroscopic appearance of samples.

A contact angle measuring instrument (JC2000D2, Shanghai zhongchen digital technic apparatus Co. Ltd, Shanghai, China) was used to test the wetting angle of the Sn-3Ag solder balls (diameter 760 μm, 1.6 mg weight/ball, Langfang Bangzhuang Electronic Materials Co. Ltd, Langfang, China) on the sapphire with and without metallization in an atmospheric environment at 240 °C. Afterwards, sandwich-structured Cu/solder/sapphire solder joints were prepared by the reflow process. Pure copper substrates (purity 99%, 5 mm × 5 mm × 1 mm) and nickel-plated sapphire substrates were used as the upper and lower substrates, while solder pastes were evenly plated on the sapphire surface by a 100 μm thick stainless mask to connect the substrates. Then, the assembled Cu/solder/sapphire sandwich structure was prepared in a crucible furnace (vacuum degree <10^−3^ Pa) at a peak reflow temperature of 250 °C for 60 min under a pressure of 0.2 Mpa. The macroscopic appearance of sapphire solder joints is shown in Figure 1. The interfacial microstructure, morphology and element distribution of the sapphire solder joints and shear fracture surface were observed by SEM and EDS.

## 3. Results and Discussion

### 3.1. Metallization Mechanism on Sapphire

The surface activity of sapphire is rather poor and cannot auto catalyze, so the pretreatment is very important for deposition on the sapphire surface. The pretreatment process mainly includes surface degreasing, polishing, chemical etching, chemical sensitization and activation. The roughness of the polished and smooth surface measured by atomic force microscope (AFM) was 84.0 nm and 1.79 nm, respectively. Some works show that substrates with reasonable surface roughness can provide good anchorage for Ni-P films, which can significantly improve deposition adhesion [19,20]. After grinding, the deposition area between the coating and sapphire is greatly increased, thereby improving the bonding force of the deposit. Chemical treatment is also carried out to further improve the bonding force. Since the substrate is α-Al_2_O_3_, which is the most stable phase in the majority of alumina and is generally insoluble in acids and alkalis. In the subsequent electroless plating process, a mixed solution of HF and HCl was used as the coarsening solution. Therefore, Ni-P coating can be deposited on the sapphire, indicating that the coarsening solution can react with α-Al_2_O_3_ and form enormous micro-holes on the substrates. Thus, the substrate can be activated for the following electroless plating. During the plating process, a complete Ni-P coating can be directly deposited on the sapphire surface at once. The results show that sapphire can be metallized by a simple electroless plating process with proper pretreatments.

**Figure 2:**
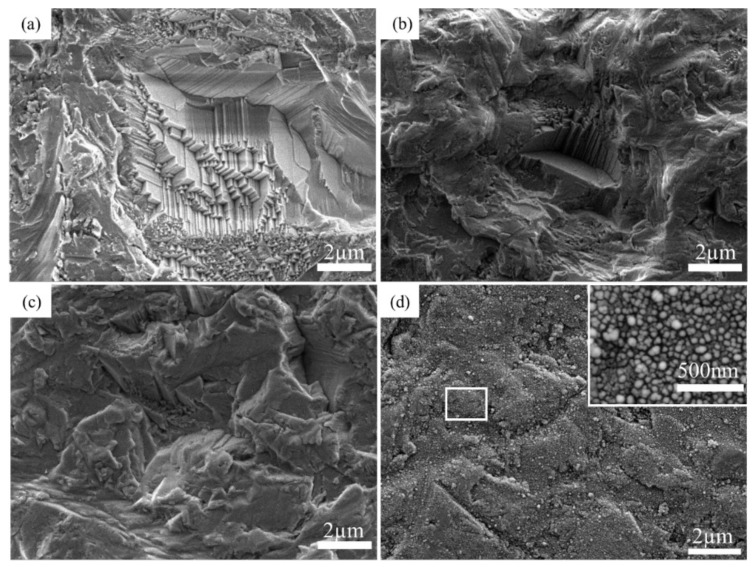
Topography of the substrate in electroless plating pretreatment after (**a**) grinding, (**b**) etching, (**c**) activation and (**d**) initial plating.

**Figure 3:**
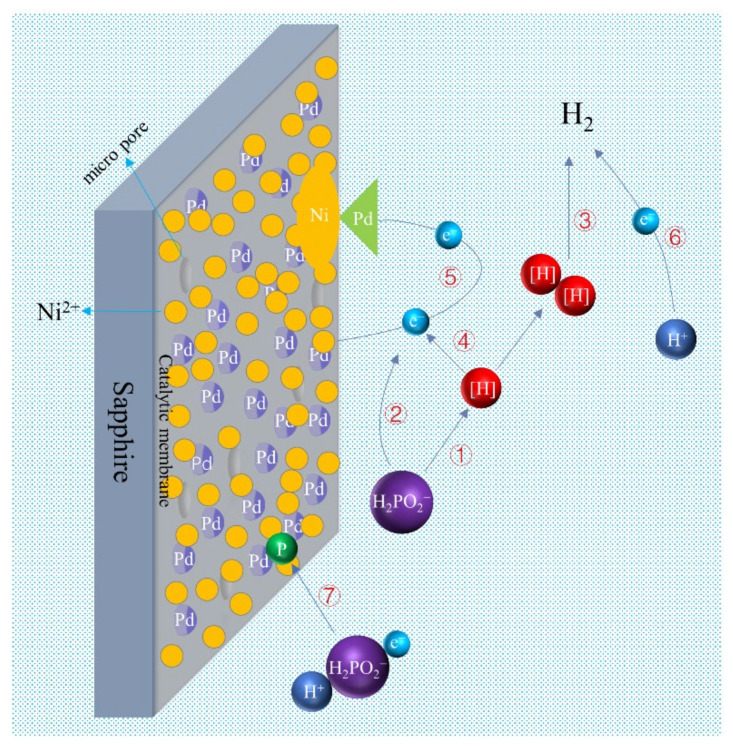
Deposition process and mechanism in the electroless plating solution.

Figure 2 shows the surface morphology of sapphire substrates in each pretreatment process. Figure 2a–d is the substrate surface after grinding, etching, activating and initial plating, respectively. Figure 2a,b has a similar microstructure, while the surface morphology in Figure 2b is smoother. This is contributed to the etching solution that can react with the sapphire substrates. Figure 2c shows the morphology of sapphire substrates after activation by SnCl_2_/HCl and PdCl_2_/HCl solution. When the specimens absorbed with *Sn^+2^* ions were immersed in the dilute PdCl_2_ solution, the *Pd^+2^* ion was reduced to Pd metal and acted as a catalyst to initiate the deposition reaction according to Equation (1). As a result, some individual *Pd* patches can be formed on the sapphire surface, as shown in Figure 3.

(1) $$Sn^{+2}+Pd^{+2}\to Sn^{+4}+Pd\;$$

Figure 2d exhibits the surface morphology of the sapphire after pre-plating for 25 s. As can be seen from the high-magnification figure in Figure 2d, enormous small particles of the Ni-P alloy are deposited on the sapphire surface in uniform distribution. Generally, the size range of Ni-P particles is between 50 nm to 100 nm. No area that is not covered with Ni-P coatings can be found even after electroless plating for only 25 s, indicating this work has a relatively fast deposition rate. The results show that by changing the pretreatment, the coating can be deposited on the sapphire substrate, and the metallization of sapphire is successfully realized.

To further explore the deposition mechanism of the sapphire metallized layer, Figure 3 is the reaction diagram simulating the Ni-P depositions on sapphire in the electroless plating solution. Under acidic conditions, the electroless nickel plating process of the phosphite ester system is basically carried out according to the following equation [21,22]. As a reducing agent, sodium hypophosphite first undergoes a dehydrogenation reaction, and the phosphorus–hydrogen bond is split to produce HPO_2_^−^ ions and reducing hydrogen atoms, as shown in Equation (2). However, HPO_2_^−^ will be oxidized to HPO_3_^−^ due to instability, and electrons will be released at the same time according to Equation (3). The reducing hydrogen atoms are oxidized in the acid solution to generate electrons (Equation (4)). Since there are individual catalytic Pd patches on the sapphire surface, Ni^2+^ will accumulate on the sapphire substrates in a large amount and be reduced to metal after obtaining nickel electrons according to Equation (5). At the same time, the hypophosphite also obtains electrons and is reduced to elemental phosphorus (Equation (6)). Since metallic nickel and elemental phosphorus are produced by the reaction at the same time and then deposited on the sapphire substrates. Thus, the deposition of the Ni-P coating can be realized. Meanwhile, many bubbles were generated during the electroless plating process. These bubbles are reduced to H_2_ by the combination of two reducing hydrogen atoms or electrons obtained from hydrogen ions in Equations (7) and (8).

(2) $$H_{2}PO_{2}^{-}\;\to \;HPO_{2}^{-}\;+\;\left[H\right]\;$$

(3) $$HPO_{2}^{-}+\;H_{2}O\;\to \;H_{2}PO_{3}^{-}\;+\;H^{+}\;+\;e^{-}$$

(4) $$\left[H\right]\to \;H^{+}\;+\;e^{-}$$

(5) $$Ni^{2+}\;+\;2e^{-}\;\to \;Ni$$

(6) $$H_{2}PO_{2}^{-}\;+\;2H^{+}\;+\;e^{-}\;\to \;2H_{2}O\;+P$$

(7) $$2H^{+}\;+\;2e^{-}\;\to \;H_{2}$$

(8) $$\left[H\right]+\;\left[H\right]\to \;H_{2}$$

### 3.2. Microstructure and Wettability of Ni-P Coatings

**Figure 4:**
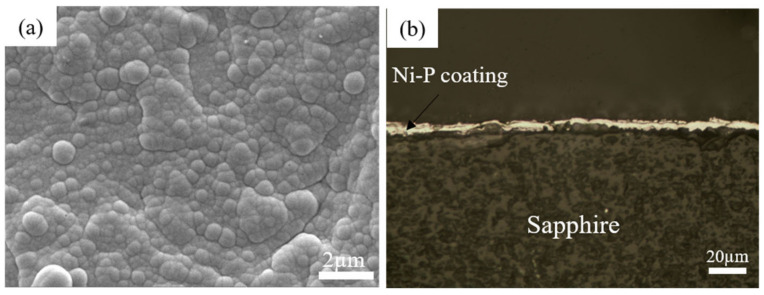
(**a**) Surface morphology, (**b**) section morphology of Ni-P coating.

The surface metallization on sapphire by electroless plating was successfully achieved. Figure 4a shows the surface morphology of the Ni-P coating by scanning electron microscope (SEM). No defects, including micropores, cracks and undeposit areas, can be found in the coating, which shows good quality. This coating exhibits a nodulus structure with a particle range of 0.45–0.96 µm. Figure 4b shows the cross-section topography of the Ni-P coating. The middle bright layer is the Ni-P coating with a relatively uniform thickness of 6.3 µm. Generally, this metallized layer is evenly distributed on sapphire and shows good quality. 

**Figure 5:**
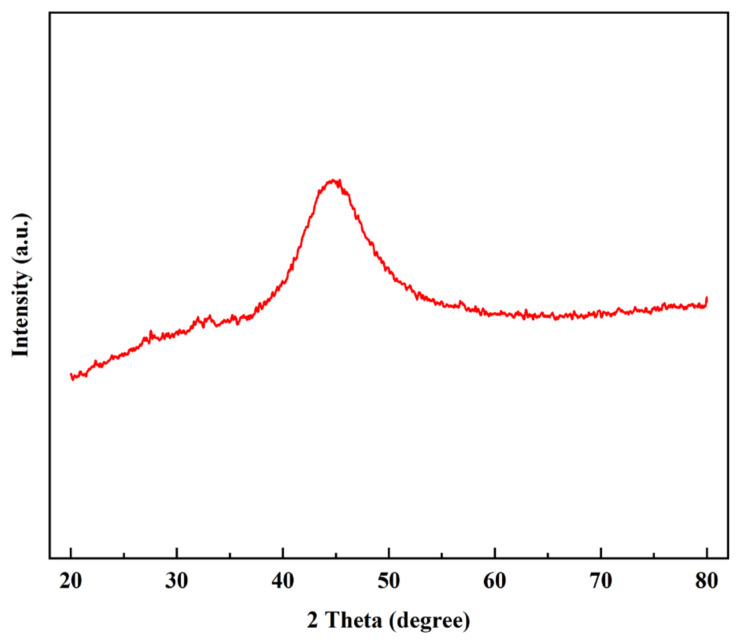
XRD pattern of Ni-P coating.

Figure 5 shows the XRD pattern of Ni-P coating, and there is a wide peak of Ni phase at 46°. In addition, the crystallinity of this coating is 0% calculated by JADE software, indicating that this coating has an amorphous structure. Moreover, the P content of this Ni-P coating is around 13–14 wt.%. As previously reported, the lattice strain of Ni (111) grains increases when the P content in Ni-P alloy exceeds 8 wt.%, which leads to a Ni-P coating with an amorphous structure [23]. 

**Figure 6:**
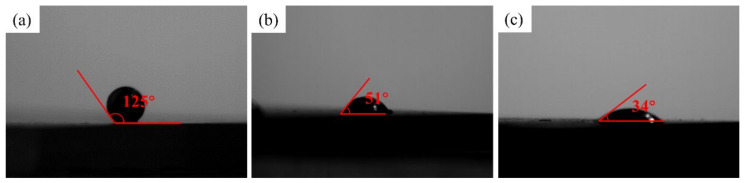
Wetting angles of Sn-Ag solder balls on (**a**) bare sapphire, (**b**) Ni-P plated sapphire and (**c**) bare Cu substrate.

Moreover, the vital effect of depositions on sapphire is to improve its solderability. Figure 6a,b are the cross-sections of the wetting behaviors of the Sn-3 Ag solder balls on sapphire without and with metallization, respectively. According to the Young’s equation [24], the solid–liquid interaction occurring when a droplet contacts a surface is expressed by the following equation:

(9) $$\cos\theta =\frac{\gamma_{sg}-\gamma_{sl}}{\gamma_{gl}}$$

where *θ* is the contact angle of a smooth surface; *γ_sg_*, *γ_sl_* and *γ_gl_* represent the interfacial tension at solid-gas, solid–liquid and gas–liquid interfaces, respectively. 

Wettability is the ability of liquid solders that spread on the surface to be soldered. Many wettability studies have shown that the values of the wetting angle can indicate the degree of wettability, as shown in Table 3 [25]. The contact angle of Sn-Ag solder on a bare copper substrate is 34°, as shown in Figure 6c, which shows good wetting. The contact angle of the sapphire sample without the Ni-P coating is 125°, which exceeds 90° and exhibits unacceptable wettability for Sn-based solder balls. On the contrary, with the aid of Ni-P coatings on sapphire, the contact angle can significantly decrease to 51°, enhancing wettability. 

### 3.3. Joining and Fracture Mechanism of Sapphire Solder Joints

**Figure 7:**
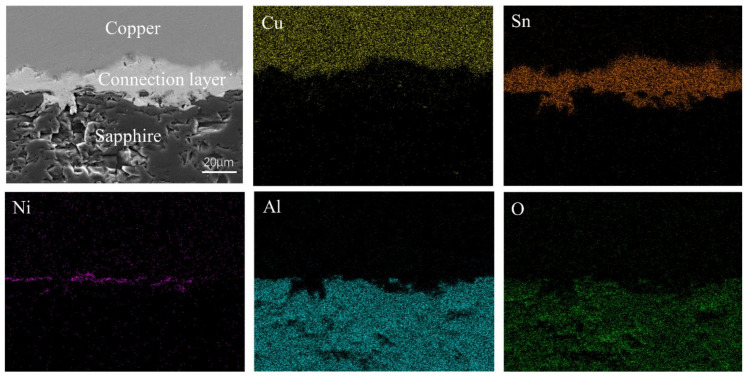
Morphology and EDS elemental distribution at Cu/sapphire solder interfaces.

Afterwards, sapphire substrates plated with Ni-P coatings were joined with bare copper sheets by a simple reflow process. Figure 7 shows the surface topography of the sapphire solder joints and the elemental distribution. The interface microstructure at the Cu/sapphire solder interfaces shows a dense structure without obvious holes and cracks. From the EDS mapping results, the bright gray stripe in the middle is the Sn-based solder, while the upper and lower substrates are copper and sapphire, respectively. Combined with the overall morphology and Ni distribution, it is found that there is an intermittent Ni-P coating between the middle layer and the sapphire, and the coating is deposited on the sapphire with good adhesion. It shows that the coating thickness decreases gradually during the reflow process, which is caused by the reaction between the Ni-P coating and the solder. 

**Figure 8:**
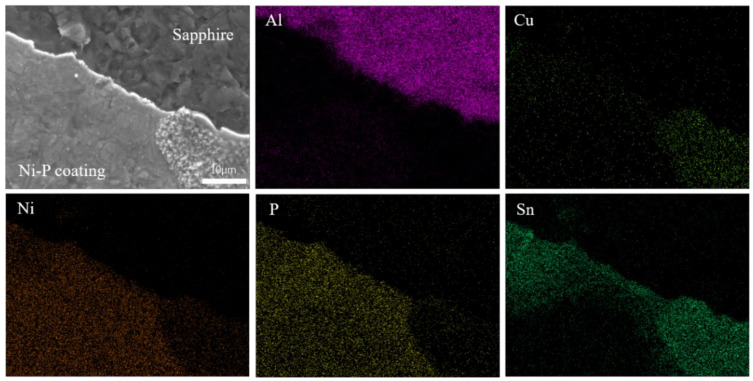
Element analysis diagram of fracture surface morphology.

The fracture surface topography and EDS elemental distribution are shown in Figure 8. It can be seen that there is an obvious bright white curve in the middle, and shear fracture mainly occurs at the interface between the coating and the substrate. The distribution of Sn and Ni indicates that the electroless nickel plating layer is reacted by metallurgy during the joining process. It is speculated that Sn-Ni intermetallic compounds are formed, which is consistent with studies on the interfacial reaction between Ni-P coatings and Sn-based solders [26,27].

**Figure 9:**
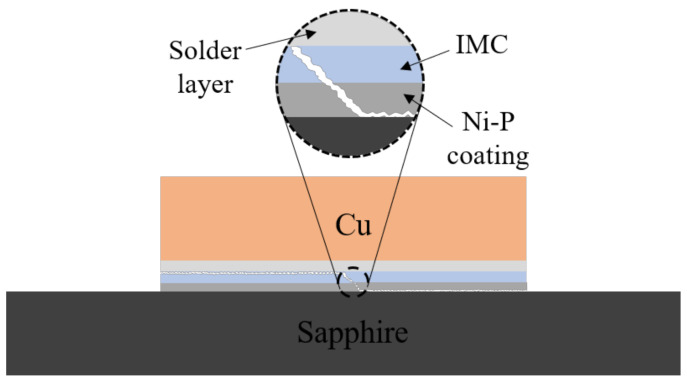
Cu/solder/sapphire fracture schematic diagram.

The fracture diagram of these sapphire solder joints is illustrated in Figure 9. During the connection process, the Sn-based solder reacts with the Ni-P coating to form Ni-Sn IMC layers, leading to a continuous decrease in the thickness of the Ni-P coating. When the thickness of Ni-P coating decreases to a certain value, shear fracture occurs at the coating–sapphire interface. 

## 4. Conclusions

In this work, the sapphire substrates were successfully catalytic activated and metallized by electroless nickel plating to achieve direct joining with copper substrates via the conventional reflow process. The conclusions can be drawn as follows:

1. A proper pretreatment for sapphire substrates was proposed to successfully deposit metallization on sapphire by a simple electroless plating process. Moreover, the deposition mechanism of the metallization on sapphire was also elaborated.
2. The metallization significantly improved the wettability of the substrates as the wetting angle of the Sn-based solders on sapphire with the metallization was reduced from 125° (without metallization) to 51°.
3. The direct joining between sapphire and copper was achieved by the conventional reflow process at 250 °C and reduced pressure. The nickel atoms from the Ni-P metallization interacted with Sn atoms in the solder, resulting in the formation of Ni-Sn IMCs to achieve metallurgical bonding at the joint interfaces.

## Figures and Tables

**Table 1:** Studies on metallization methods on sapphire.

Methods	Temperature	Conditions	Disadvantage	Reference
Mo-Mn sintering	950~1000 °C	Reducing atmosphere	Pore and microcrack defects	[6]
Magnetron sputtering	<50 °C	Vacuum condition	High cost, low material utilization	[7]
Ultrasonic assisted hot dipping	210~230 °C	Active solders (Ti, Al, Mg)	Ultrasonic damage	[8,9]

**Table 2:** Chemical plating bath composition and conditions.

Chemical Reagents	Composition (g/L)
NiSO_4_·6H_2_0	20–35
NaH_2_PO·2H_2_O	25–35
Na_3_C_6_H_5_O_7_·2H_2_O	60–70
((NH_4_)_2_SO_4_	60–80
Plating condition	Temp: 65–75 °C, pH: 5–6, Time: 15–30 min

**Table 3:** The correlation of wetting angle range with wettability.

Wetting Angle Range	Wettability
0° ≤ *θ* < 30°	Excellent wetting
30° ≤ *θ* < 40°	Good wetting
40° ≤ *θ* < 55°	Acceptable wetting
55° < *θ* < 70°	poor wetting
70° ≤ *θ*	Unacceptable wetting

## Data Availability

Data presented in this article are available at request from the corresponding author.
